# Long-term results of ERCP- or PTCS-directed photodynamic therapy for unresectable hilar cholangiocarcinoma

**DOI:** 10.1007/s00464-020-08095-1

**Published:** 2020-10-26

**Authors:** Zongyan Li, Xiaofeng Jiang, Hua Xiao, Shaoyi Chen, Wenfeng Zhu, Haiwu Lu, Liangqi Cao, Ping Xue, Haiyan Li, Dawei Zhang

**Affiliations:** 1 Department of Hepatobiliary Surgery, The Second Affiliated Hospital of Guangzhou Medical University, #250 East Changgang Road, Guangzhou, 510260 China; 2 Department of General Surgery, Tongji Huangzhou Hospital of Huazhong University of Science and Technology, Wuhan, China; 3 Department of Breast Surgery, The Sixth Affiliated Hospital of Sun Yat-sen University, Guangzhou, China

**Keywords:** Cholangiocarcinoma, Photodynamic therapy, ERCP, PTCS

## Abstract

**Background:**

Photodynamic therapy (PDT) can be performed as palliative therapy for cholangiocarcinoma, while there is currently insufficient evidence for the efficacy. The aim of this study was to explore the clinical efficacy and safety of endoscopic retrograde cholangiopancreatography (ERCP)- or percutaneous transhepatic cholangioscopy (PTCS)-directed PDT combined with stent placement for unresectable hilar cholangiocarcinoma.

**Methods:**

A retrospective analysis was conducted on 62 patients with unresectable hilar cholangiocarcinoma. Thirty patients received PDT using hematoporphyrin combined with biliary stent placement (PDT+stent group), including 22 receiving ERCP-directed PDT and 8 receiving PTCS-directed PDT. Survival time, quality of life, and postoperative adverse events were compared to 32 patients receiving biliary stent placement alone (Stent-only group).

**Results:**

After 42 months of follow-up, median survival time was significantly longer in the PDT+stent group than the Stent-only group (14.2 vs. 9.8 months, *P* = 0.003). In the PDT+stent group, the median survival time was longer in the 6 patients with recurrence after surgical resection than the 24 patients without prior surgical resection (20.0 vs. 13.0 months, *P* = 0.017). The QOL total scores was significantly higher in the PDT+stent group than the Stent-only group at postoperative 6, 9, and 12 months (*P*<0.05). There was no significant difference in the incidence of postoperative adverse events between the two groups (24 [38.7%] vs. 20 [29.0%], *P* = 0.239).

**Conclusion:**

ERCP- or PTCS-directed PDT + stent placement can prolong the survival of patients with unresectable hilar cholangiocarcinoma, especially those with recurrence and improve quality of life without increasing adverse events.

Most patients with hilar cholangiocarcinoma are ineligible for surgery [1], endoscopic biliary stent placement can relieve jaundice and improve quality of life, but tumor progression is not controlled and the survival time is limited [2, 3]. Photodynamic therapy (PDT) is a relatively new targeted tumor ablation in which patients are injected with a photosensitizer that accumulates preferentially in tumor tissues and can destroy local tissue via reactive oxygen species (ROS)-induced toxicity upon excitation at the appropriate excitation wavelength [4]. Recent animal experiments have shown that PDT can also produce anti-tumor immune responses following initial ROS-mediated damage for better therapeutic effect [5–7].

Recent studies have shown that PDT for unresectable cholangiocarcinoma can reduce bile duct stenosis, improve quality of life, and prolong survival [8–10]. In fact, there are now clinical guidelines or specifications that recommend PDT as one option for palliative treatment of unresectable cholangiocarcinoma, including Hepatobiliary Cancers, Version 2.2014 published in 2014 by the National Comprehensive Cancer Network (NCCN) [11], and the Asia-Pacific Consensus Recommendations published in 2013 [12].

PDT has also been explored for patients with local post-surgical recurrence of hilar cholangiocarcinoma. Shimizu et al. [13] reported one patient whose recurrent lesion at the site of cholangioenteric anastomosis disappeared one week after PDT, and no tumor residue was found at one-month follow-up. At present, however, there are few clinical studies on PDT for postoperative recurrence of hilar cholangiocarcinoma and little long-term follow-up data supporting curative effects. Nonetheless, the few exploratory results reported have suggested promise for hilar cholangiocarcinoma.

PDT is mainly achieved by introducing an optical fiber into the tumor site under either ERCP or PTCS guidance. The current study evaluated the efficacy and safety of ERCP- or PTCS-directed PDT for treatment of unresectable hilar cholangiocarcinoma, including some cases of post-surgical recurrence.

## Methods

This was a retrospective cohort study between October 2008 and August 2012. Among the 122 patients, those meeting the following conditions were included in this study: (1) male or female aged 18–75 years, (2) with intrahepatic bile duct dilatation and hilar bile duct space-occupying lesions detected by magnetic resonance cholangiopancreatography (MRCP), (3) diagnosed by cytology brush or tissue biopsy, (4) no previous chemoradiotherapy, (5) providing signed informed consent, and (6) meeting criteria for unresectable hilar cholangiocarcinoma: (a) Bismuth-Corlette type IV [14] ineligible for liver transplantation, (b) TNM Stage III and Stage IV [15], (c) recurrent hilar cholangiocarcinoma after radical resection, (d) surgical contraindications independent of Bismuth type and TNM stage. This study was performed in accordance with the Declaration of Helsinki. The Strengthening the Reporting of Observational Studies in Epidemiology (STROBE) statement recommendations were followed in the design of the study.

### ERCP technique

**Fig. 1:**
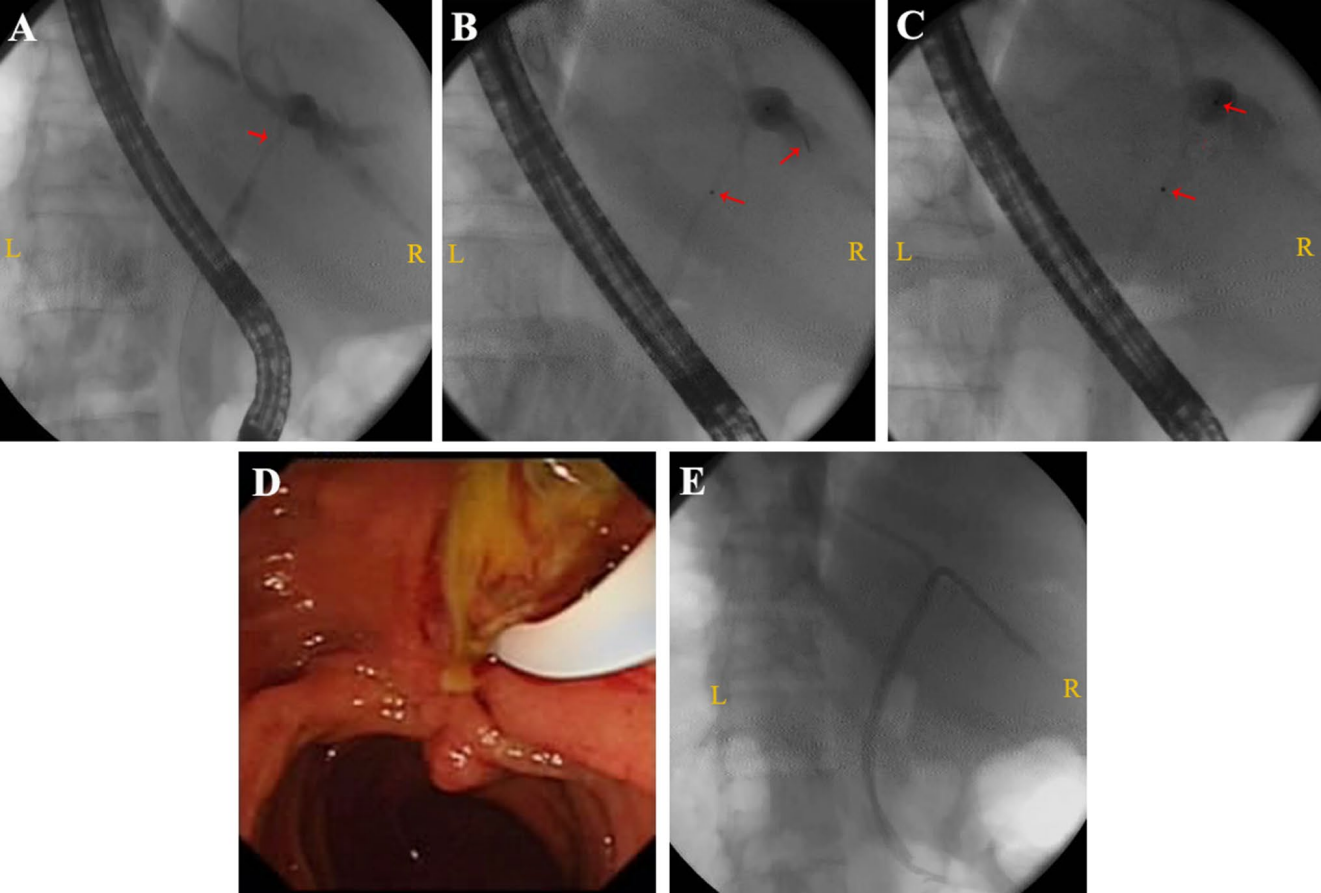
ERCP-directed PDT procedure for hilar cholangiocarcinoma. **A** Cholangiography showing the location of tumor stenosis. **B** As directed by the sphincterotomy knife, the fiberoptic columnar diffuser was positioned at the site of tumor stenosis (arrows indicate the marker of the sphincterotomy knife head end and the columnar diffuser). **C** The sphincterotomy knife was withdrawn, the fiberoptic column diffuser was left in place (arrow), and PDT irradiation was initiated. **D** Two months post-treatment, a balloon was used to remove necrotic and exfoliated tissue to clear the bile duct. **E** Two plastic biliary stents were placed to ensure biliary drainage

The cytological examination was conducted under ERCP, when diagnosis of cholangiocarcinoma was confirmed, PDT directed by ERCP was conducted again. The operator delivered the duodenoscope (TJF-140, TJF-160VF, Olympus, Tokyo, Japan) to the duodenal papilla, and any previously placed biliary stent or nasobiliary drainage catheter was removed first. A sphincterotomy knife (Boston Scientific, Marlborough, USA) was then directed by a guidewire from the duodenal papilla cannula into the tumor stenosis position as shown by angiography. In case of severe tumor stenosis, a biliary dilatation catheter (Cook Medical, Bloomington, USA) was used to guide and deliver the PDT cylindrical optical fiber to the location of stenosis as directed by the sphincterotomy knife. A metal marker was attached to the head end of the cylindrical optical fiber for X-ray localization. The sphincterotomy knife or dilatation catheter was then withdrawn and the optical fiber was retained in the original position for irradiation. The necrotic and exfoliated tissues were removed with a balloon to restore patency of the bile duct, and the optical fiber was replaced with a guidewire and plastic stents (Fig. 1). The ERCP was performed by 1 of 3 experienced hepatobiliary endoscopic surgeons. All three conduct more than 200 ERCP procedures per year.

### PTCS technique

**Fig. 2:**
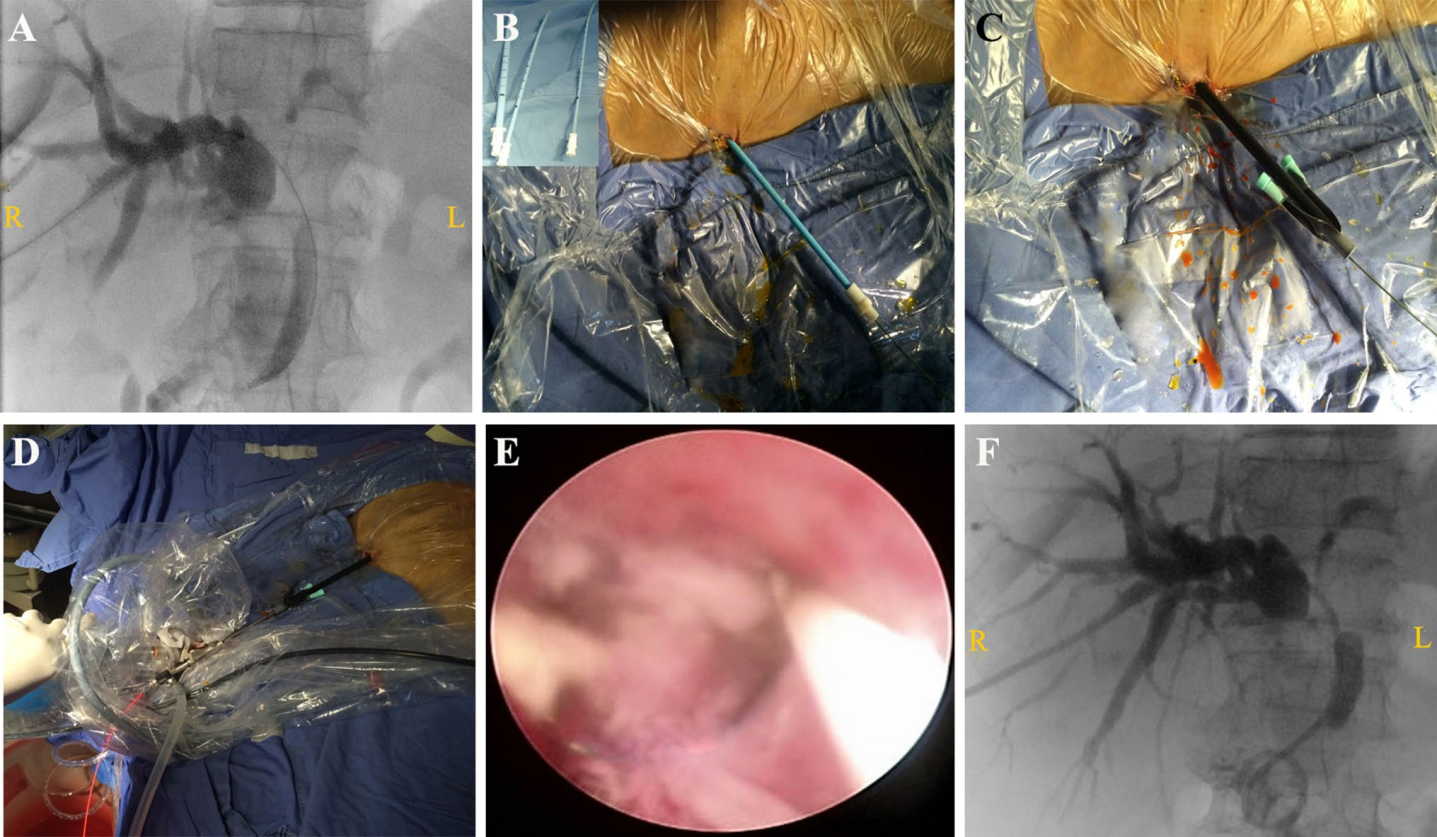
PTCS-directed PDT procedure. **A** Percutaneous transhepatic cholangial drainage. Cholangiography showing the location of tumor stenosis. **B**, **C** As directed by a rigid guidewire, the skin and bile duct were expanded step-by-step using a percutaneous dilatation tube, and the 16Fr sheathing canal was retained. **D**, **E** After inserting the rigid choledochoscope from the sheathing canal, the tumor was visualized directly under choledochoscopy, and the fiberoptic column diffuser was placed at the tumor location for PDT. **F** Internal and external drainage catheter were placed in the bile duct to ensure biliary drainage

The PTCS procedure was conducted in two stages. In the first stage, percutaneous transhepatic cholangial drainage (PTCD) was performed and biliary cholangiography was used to locate the tumor stenosis. Cytological examination was performed and the internal and external biliary drainage catheters (Cook Medical, G09497) were retained. When cytological diagnosis was confirmed, PTCS-directed PDT was performed again. A percutaneous dilatation catheter (Amplatz renal introducer, G18013, Cook Medical) directed by a rigid guidewire was used for step-by-step dilation, starting from 8 French (Fr), gradually expanding at an interval of 2Fr up to 16Fr, and retaining a sheath of 16Fr. The choledochoscope was fed through the sheathing canal to directly visualize the tumor. After biopsy, the cylindrical optical fiber was positioned for PDT. At the end of PDT, the drainage catheters were inserted (Fig. 2).

### Photodynamic therapy

Hematoporphyrin (Huading Modern Biopharmaceutics Co., Ltd., Chongqing, China) was used as the photosensitizer. Patients were administered 2.0 mg/kg in 250 ml of 5% glucose solution by photophobic intravenous drip at 60 drops/min. Irradiation was applied 48 h after administration. A PDT optical fiber (LG-PDT-02 PDT Laser, 3 m in total length and 400 μm in core diameter, plus a cylindrical diffuser of 20–50 mm length equipped with a X-ray marker at the head end, Leigao Medical Instrument Co., Ltd., Chongqing, China) was introduced into the location of tumor stenosis. Excitation light at 630 nm was delivered for 25 min at a dose of 250 J/cm^2^. All patients were advised to stay out of direct natural light for 2 weeks after PDT. PDT was performed at an interval of two months when the patient was in good physical condition.

### Stent placement

The plastic biliary stents (Boston Scientific) of 7.0 or 8.5 Fr in diameter were inserted after PDT directed by ERCP, with the number according to the Bismuth type of cholangiocarcinoma. Bilateral stents were placed for type IV, while one stent was placed for types IIIA and IIIB. If indicated by PTCS, the biliary drainage catheters (COOK Medical) of 8.5Fr in diameter were inserted. Two drainage catheters for type IV and one catheter for types IIIA and IIIB were placed.

### Adverse events

The most frequent PDT-related adverse events are skin phototoxic reactions. Common ERCP- and PTCS-related adverse events include acute cholangitis, pancreatitis, liver abscess, hemobilia, and duodenal perforation. Acute cholangitis was defined as fever (body temperature > 38.5 °C) and chills accompanied by elevated bilirubin. Pancreatitis was defined as abdominal pain and elevated serum amylase.

### Follow-up

Patients were followed up every 3 months from first operation until death by outpatient visit or by e-mail or telephone. Early withdrawals were excluded from the final analyses. The last follow-up visit was conducted in February 2016. All patients underwent laboratory tests one and two months after the operation and every three months thereafter. Quality of life was assessed by the FACT-HEP scale [16]. Survival time was defined as the interval between the first PDT or stent placement and death.

### Statistical analysis

Categorical variables were analyzed using the Chi-squared test or the Fisher exact test; continuous variables were analyzed using Student’s *t* test or non-parametric tests. Survival time was estimated by a Kaplan-Meier method and compared between groups with the log-rank test. Multivariate survival analysis was conducted by using a Cox proportional hazards model. In addition to survival analysis between patients with postoperative recurrence and unresectable patients, the statistical significance was adjusted to 0.025, other statistical significance was assumed to be at an alpha of 0.05, all statistical tests were two-sided. All statistical analyses were performed using SPSS version 16.0 (SPSS, Chicago, Illinois, USA).

## Results

### Patient characteristics

**Fig. 3:**
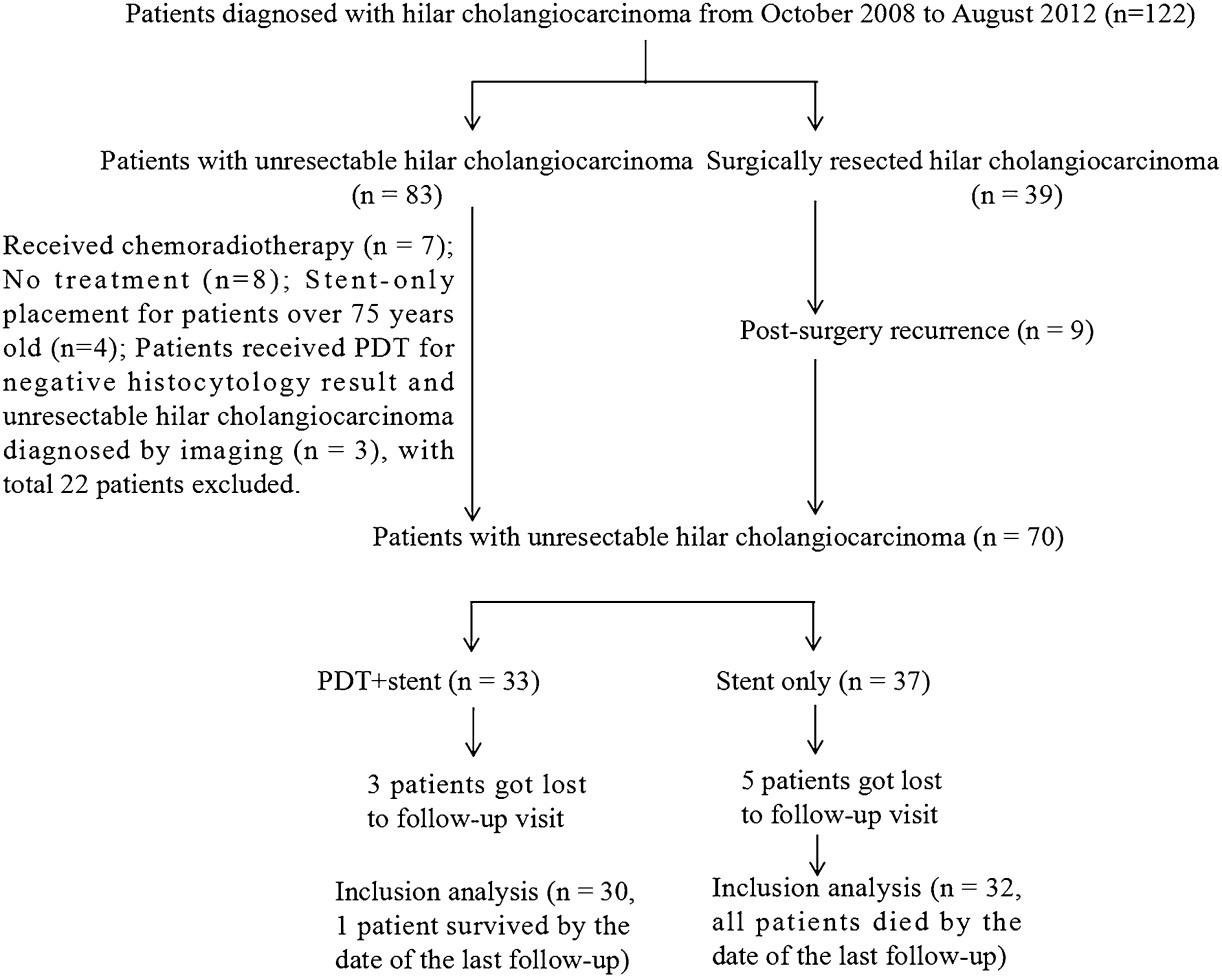
Flow chart showing participant selection and exclusion

From October 2008 to August 2012, 122 patients with obstructive jaundice and diagnosed with hilar cholangiocarcinoma were treated at our institution, of which 62 met study inclusion criteria. Among these, nine showed recurrence (Fig. 3). All patients were confirmed by histology or cytology. Thirty patients received PDT + stent placement (the PDT + stent group), 22 patients by ERCP-directed PDT and 8 by PTCS-directed PDT, among these eight patients, six patients showed recurrence, one patient with a history of subtotal gastrectomy and one with unsuccessful ERCP operation. Alternatively, 32 patients received biliary stent placement only (Stent-only group), of which 26 were directed by ERCP and 6 received percutaneous transhepatic cholangial drainage (PTCD) (including three patients with recurrence and three patients with unsuccessful ERCP operation). There were no significant differences in mean age, sex ratio, preoperative bilirubin level, Bismuth type, TNM stage, recurrence rate, PDT guidance methods, and FACT-HEP scores between two groups (Table 1).

**Table 1:** Baseline characteristics of patients with unresectable cholangiocarcinomas

	PDT + stent group n = 30	Stent-only group n = 32	*P* value
Age, mean ± SD, years	53.8 ± 12.2	54.7 ± 10.7	0.78^a^
Sex, male/female, *n*	12/18	10/22	0.47^b^
Preoperative bilirubin, mean ± SD, mg/dL	28.1 ± 7.3	30.5 ± 6.4	0.17^a^
Bismuth type, *n*			
	6	9	0.55^b^
	24	23	
TNM stage, *n*			
	4	8	0.25^b^
	26	24	
Recurrence after resection, *n*	6	3	0.14^b^
Approach procedure to the stenosis , *n*			
ERCP-directed	22	26	0.64^b^
PTCS-directed	8	6	
PDT sessions, *n*	1 × 11. 2 × 10.3 × 5.4 × 4		
FACT-HEP scores, mean ± SD	116.4 ± 5.8	115.2 ± 5.5	0.49^a^

*SD* standard deviation; *PDT* photodynamic therapy; *ERCP* endoscopic retrograde cholangiopancreatography; *PTCS* percutaneous transhepatic cholangioscopy; *FACT-HEP* Functional Assessment of Cancer Therapy-Hepatobiliary questionnaire

^a^Student’s *t* test

^b^Chi-squared test

### Survival analysis

**Fig. 4:**
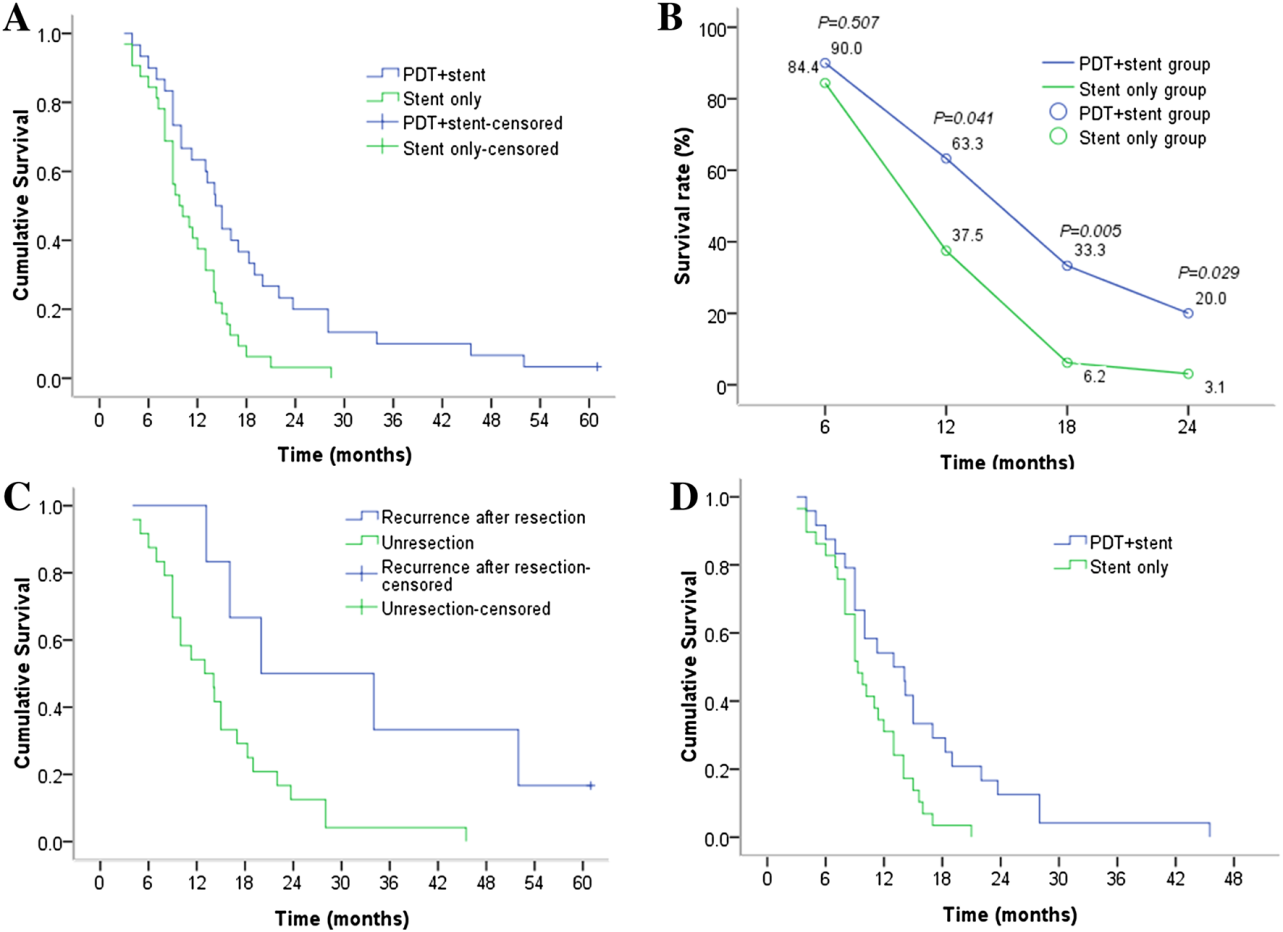
Kaplan–Meier survival curves of the study patients. **A** Comparison of survival times between the PDT + stent group and Stent-only group. **B** Comparison of survival rates between PDT + stent group and Stent-only group at different post-treatment time points. **C** Comparison of survival times between patients with recurrence after surgical resection and patients without surgical resection in the PDT + stent group. **D** Comparison of survival times between patients with non-postsurgery recurrence from the PDT + stent and Stent-only groups

Only one patient (in the PDT + stent group) survived until the final follow-up in February 2016. Median survival time was longer in the PDT+stent group than the Stent-only group (14.2 months, 95% confidence interval [CI] 11.8–16.6 vs. 9.8 months, 95% CI 7.0–12.6, *P* = 0.003). Compared to the Stent-only group, the PDT+stent group also demonstrated significantly ($P<$ 0.05) greater survival rates at 12 months (63.3% vs. 37.5%), 18 months (33.3% vs. 6.2%), and 24 months (20% vs. 3.1%), but not at 6 months (90.0% vs. 84.4%; *P* = 0.507). Multivariate COX regression analysis revealed that recurrence after surgical resection and PDT were protective factors prolonging survival (Table 2). In the PDT+stent group, the 6 patients with recurrence after surgical resection achieved longer median survival than the 24 patients without surgical resection (20.0 months, 95% CI 0–41.5 vs. 13.0 months, 95% CI 8.0–18.0; *P* = 0.017, adjusted test level $\alpha {}$ = 0.025. To assess survival in the absence of recurrence, non-recurrent patients in the PDT+stent and Stent-only groups were compared, which also revealed longer median survival time in the PDT+stent group (13.0 months, 95% CI 8.0–18.0 vs. 9.3 months, 95% CI 7.7–10.9, *P* = 0.01) (Fig. 4).

**Table 2:** Multivariate analysis of factors suspected to affect survival

Predictor factor	Hazard rate	95% Confidence interval		*P* value
		Lower	Upper	
Bismuth type	0.716	0.705	2.409	0.398
TNM stage	1.140	0.359	1.352	0.286
ERCP- or PTCS-directed	0.012	0.364	2.468	0.912
Recurrence after resection	5.335	1.238	13.613	0.021*
PDT	7.811	1.274	3.966	0.005*

*PDT* photodynamic therapy; *ERCP* endoscopic retrograde cholangiopancreatography; *PTCS* percutaneous transhepatic cholangioscopy

*Adjusted *P* values were derived by using the Wald test, *P* < 0.05, significant difference

### Therapeutic response of cholangiocarcinoma to PDT

**Fig. 5:**
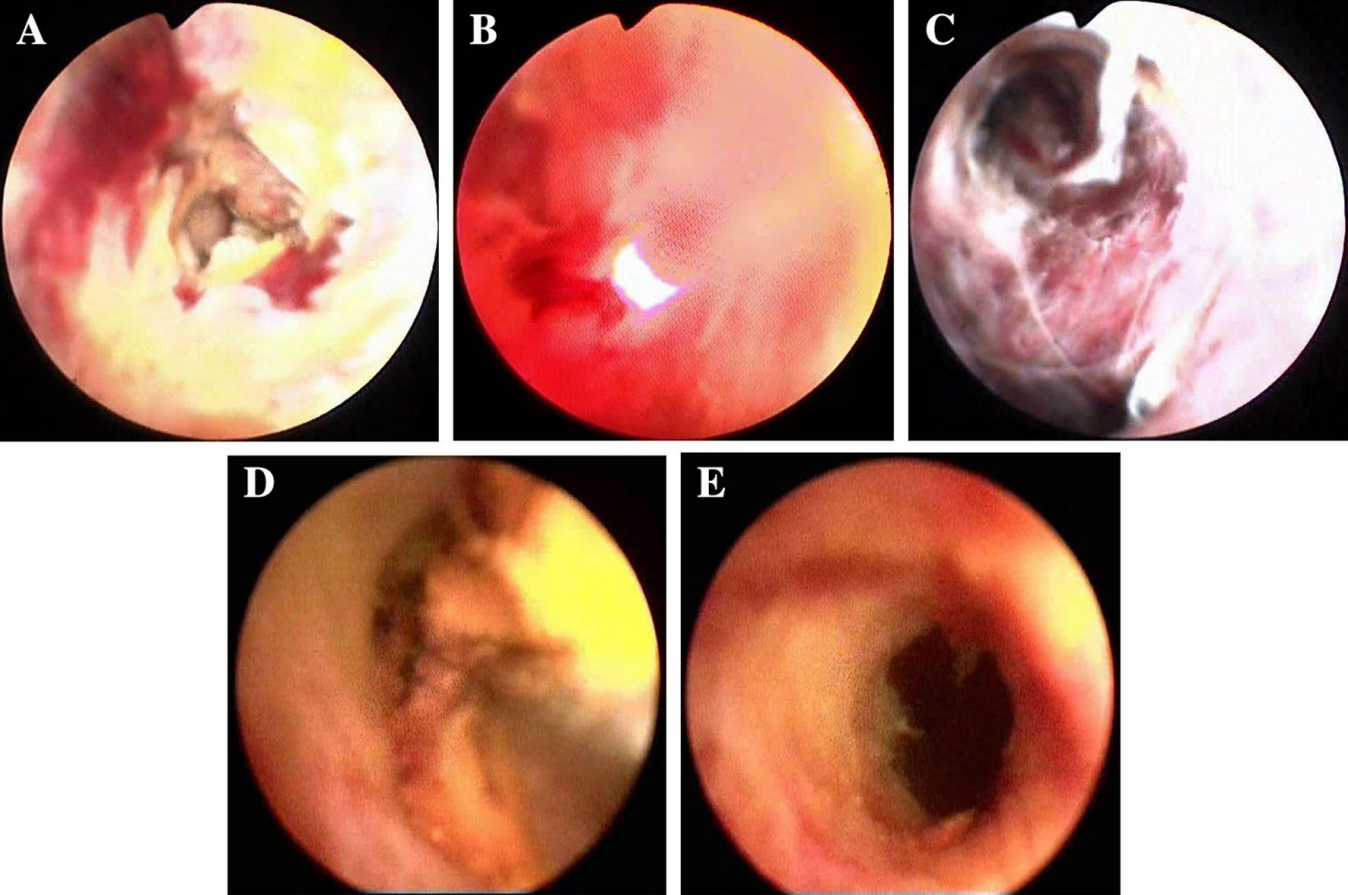
Example of therapeutic response by PTCS-directed PDT. A 56-year-old female was admitted to hospital for obstructive jaundice. Ten months previously, the patient received left hemi-hepatectomy + right hepatic duct-Jejunostomy for hilar cholangiocarcinoma (type III b). **A** Bile duct tumors were seen under choledochoscopy and confirmed by biopsy as tumor recurrence. **B** PDT under choledochoscopy 48 h after injection of the photosensitizer. **C** Choledochoscopy showing inflammatory changes in the wall of bile duct 48 h after PDT. **D** Two months after PDT, tumor necrosis and exfoliated tissue were observed in the bile duct and removed under choledochoscopy. **E** After removal of the necrotic tissue, the bile duct was unobstructed, the inflammatory reaction disappear, and the tube wall was smooth

Photodynamic therapy induced tumor tissue necrosis and relieved malignant biliary stricture as evidenced by PTCS. Bile duct obstruction caused by tumor could be observed under choledochoscopy and was confirmed as cholangiocarcinoma by tissue biopsy. Forty-eight hours after photosensitizer injection, the cylindrical diffuser of the optical fiber with choledochoscope was positioned at the tumor for irradiation. Congestion of the bile duct wall tissue, local coagulation necrosis, and other inflammatory changes were detected under choledochoscope 48 h after PDT. Two months post-treatment, necrotic abscission tissue was observed in the bile duct under choledochoscope and excised. After excision of the necrotic tissue, the bile duct was unobstructed, the inflammatory reaction disappeared, and the duct wall demonstrated a normal smooth appearance (Fig. 5).

### Analysis of quality of life

The overall scores of FACT-HEP scale did not differ between PDT + stent and Stent-only groups before treatment (116.4 vs. 115.2, *P* = 0.642) and 3 months post-treatment (134.1 vs. 131.6, *P* = 0.115), but was significantly higher in the PDT + stent group at 6, 9, and 12 months post-treatment compared to the Stent-only group (Table 3).

**Table 3:** Comparison of quality of life between the two groups with FACT-Hep questionnaire

	PDT + stent group	Stent-only group	*P* value
Pre-treatment	116.4 ± 5.8	115.2 ± 5.5	0.642
Post-treatment 3 months	134.1 ± 6.9	131.6 ± 5.8	0.115
Post-treatment 6 months	135.4 ± 6.8	126.3 ± 5.9	0.026*
Post-treatment 9 months	127.0 ± 5.5	118.1 ± 4.7	0.001*
Post-treatment 12 months	128.0 ± 6.9	114.7 ± 5.6	0.001*

*P < 0.05

### Adverse events

Adverse events were observed from the first to the last administration. In the PDT + stent group, there were nine incidences of cholangitis (14.5%), seven of pancreatitis (11.3%), one case of liver abscess (1.6%), and three incidences of hemobilia (4.8%). In the Stent-only group, there were eight incidences of cholangitis (11.6%), nine of pancreatitis (13.0%), one of liver abscess (1.4%), and two of hemobilia (2.9%). There were no serious complications such as duodenal perforation in either group (Table 4). There were four cases of skin phototoxic reactions, which were improved by prolonging the time of light avoidance and delivery of topical treatment.

**Table 4:** Comparison of adverse events between the two groups

	PDT+stent group (number of operations, *n* = 62)	Stent-only group (Number of operations, *n* = 69)	*P* value
Total adverse events, *n* (%)	24 (38.7%)	20 (29.0%)	0.239
Acute cholangitis	9 (14.5%)	8 (11.6%)	0.620
Acute pancreatitis	7 (11.3%)	9 (13.0%)	0.759
Liver abscess	1 (1.6%)	1 (1.4%)	0.939
Biliary hemorrhage	3 (4.8%)	2 (2.9%)	0.562
Duodenal perforation	0	0	
Skin phototoxicity	4 (6.5%)		

## Discussion

About 70–80% of cholangiocarcinoma patients are untreatable by radical resection at the time of diagnosis, while palliative treatments such as chemoradiotherapy are of limited benefit [17]. Recent studies have shown that PDT is applicable to hilar cholangiocarcinoma in patients who (1) cannot or will not undergo surgical resection, (2) have local residual tumor, positive resection margin, or recurrent lesions after surgical operation, and (3) can receive curative resection after neoadjuvant PDT, while initially considered non-curatively resectable.

Ortner et al. [9] conducted a randomized controlled trial to compare the effect of PDT + stent to stent-only for patients with unresectable hilar cholangiocarcinoma and found significantly better median survival time in the PDT+stent group (493 days vs. 98 days, *P*< 0.0001). Similarly, a retrospective study by Kahaleh et al. [18] including 19 patients receiving PDT + stent and 29 stent-only found longer average survival time in the PDT + stent group (16.2 months vs. 7.4 months, *P*< 0.003). A larger retrospective study by Witzigmann et al. [19] including 184 patients also found that PDT + stent prolonged median survival time compared to stent-only treatment (12.0 months vs. 6.4 months, *P*< 0.01). Thus, our findings of longer median survival time and greater survival rates at 12, 18, and 24 months post-treatment in the PDT+stent group versus stent-only group are consistent with other populations and underscore the efficacy of PDT for unresectable hilar cholangiocarcinoma.

PDT can also be used to treat post-surgical residual tumor, positive resection margin, or recurrence of cholangiocarcinoma. Nanashima et al. [20] reported the benefits of PDT in eight patients with cholangiocarcinoma after resection, including 6 with positive resection margin, one with biliary obstruction caused by residual tumor, and one with recurrence after resection. The 2-year survival rate following PDT was 75%, and four of eight patients showed no recurrence during the 20-month follow-up. In Our studies, the six patients with post-surgical recurrence achieved longer median survival time than the 24 without surgical resection (20.0 vs. 13.0 months) in the PDT + stent group, suggesting that PDT may be more effective in hilar cholangiocarcinoma patients with post-surgical recurrence.

At present, there are two major PDT methods for hilar cholangiocarcinoma, ERCP and PTCS, each with its own advantages and disadvantages. ERCP is the preferred method, but requires X-ray fluoroscopy to display the optical fiber marker at the tumor site. Alternatively, PTCS is mainly suitable for patients with post-surgical recurrence and difficulty in ERCP operation. The major advantage of PTCS is direct viewing of the tumor for more accurate localization and assessment of therapeutic response, while disadvantages include relatively greater trauma due to percutaneous transhepatic puncture. Lee et al. [21] compared the efficacy between 13 ERCP-directed PDT and 24 PTCS-directed PDT cases for treatment of advanced hilar cholangiocarcinoma and found no significant difference in median survival time (11.6 vs. 9.5 months) and median stent patency time (7.2 vs. 6.2 months). Alternatively, median hospitalization time was longer in the PTSC-directed group (63 vs. 37 days).

One additional benefit of PDT for hilar cholangiocarcinoma is the relative short response time. Ortner et al. [22] reported that 5 of 9 hilar cholangiocarcinoma patients demonstrated significant reductions in tumor size (12.70 ± 6.26 cm to 4.40 ± 3.36 cm) under choledochoscopy after receiving one PDT administration, while three showed no intraductal residual tumor after 2 PDT administration. Our study revealed inflammatory changes such as hyperemia and local coagulation necrosis under choledochoscopy 48 h after PDT, and large amounts of necrotic and exfoliated tissues two months later. After removal of these tissues, inflammatory reaction of bile duct wall rapidly disappeared. Thus, PDT can induce rapid necrosis of cholangiocarcinoma and relieve biliary obstruction.

Common adverse events after PDT include acute cholangitis, pancreatitis, hematobilia, liver abscess, and skin photosensitivity reactions. Most of these are ERCP- or PTCS-related complications, including a PDT-specific skin phototoxicity [8–10, 20]. A meta-analysis of 10 studies found a pooled odds ratio for post-intervention cholangitis in the PDT group vs. stent group of only 0.57 (95% CI 0.35–0.94) and a pooled incidence of photosensitivity secondary to PDT of only 10.51% (95% CI 6.94–14.72%), reactions that were self-limiting [23]. In the current study, there were no significant differences in the incidences of specific postoperative adverse events between the PDT + stent and Stent-only groups.

The goals of cancer treatment include not only prolonged survival and improved survival rate, but restoration of quality of life (QOL) through physical, psychological, and social recovery. Heffernan et al. [16] confirmed that the FACT-HEP shows strong reliability, validity, responsiveness, and feasibility for evaluating QOL in patients with hepatobiliary malignant tumors. In the current study, FACT-HEP scale scores were higher in the PDT+stent group at 6, 9, and 12 months post-treatment compared to the Stent-only group. Thus, PDT can improve the long-term QOL.

This study has several limitations. First, this is a single-center non-randomized controlled study with a relatively small sample size; nonetheless, results showing significantly greater long-term efficacy and equivalent safety to stenting alone are a powerful reason for further larger-scale studies on the efficacy and safety of PDT for hilar cholangiocarcinoma. Second, results demonstrated greater efficacy in patients with recurrence, but only 6 such patients were included. Again, larger sample sizes are needed to increase the number of recurrent cases and confirm this finding. Third, the number of PDT treatments differed among patients (1–4). Future studies are needed to identify the optimal number for specific patient groups.

Although PDT has obvious advantages in the treatment of cholangiocarcinoma, the existing PDT has certain limitations, which limits its clinical application to a certain extent. First, the therapeutic effect of PDT is related to the production efficiency of ROS. At present, the clinical application is mainly the first generation of photosensitizers, with low ROS production efficiency [24]. Second, the tissue depth of treatment is generally only 0.4–1.0 cm [25]. Third, the dose or amount of light must be used within a safe range, otherwise it may damage the surrounding normal tissues. In recent years, with the in-depth research on the principle of photodynamics and nano photosensitizer, PDT provides a new idea for the treatment of cholangiocarcinoma.

In conclusion, ERCP- or PTCS-directed PDT is effective and safe for the treatment of unresectable hilar cholangiocarcinoma. Results must be confirmed in a large multicenter study.
